# Impact of Solvent on the Thermal Stability of Amines

**DOI:** 10.1021/acs.iecr.2c01934

**Published:** 2022-10-19

**Authors:** Karen K. Høisæter, Solrun J. Vevelstad, Lucas Braakhuis, Hanna K. Knuutila

**Affiliations:** † Department of Chemical Engineering, NTNU, NO-7491Trondheim, Norway; ‡ SINTEF Industry, P.O. Box 4760, TorgardenNO-7465, Norway

## Abstract

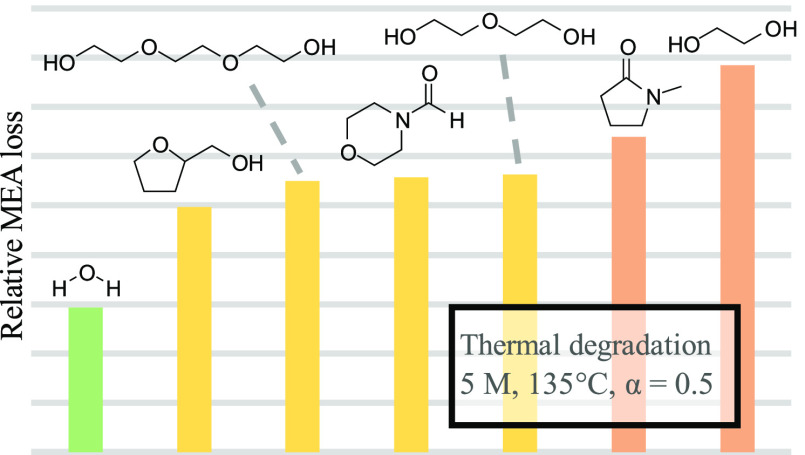

Water-lean solvents have been proposed as a possible alternative to aqueous amine systems in postcombustion carbon capture. There is however little data available on how amine degradation is affected by different solvents. This study presents new insights on the effect of solvent on thermal degradation of alkanolamines from laboratory-scale degradation experiments. Replacing the water in aqueous monoethanolamine (MEA) solutions with organic diluents resulted in varying thermal degradation rates. Overall, all tested organic diluents (triethylene glycol, diethylene glycol, mono ethylene glycol, tetrahydrofurfuryl alcohol, *N*-formyl morpholine/water, and *N*-methyl-2-pyrrolidone) resulted in higher thermal degradation rates for loaded MEA. None of the proposed parameters, such as acid–base behavior, polarity, or relative permittivities, stood out as single contributing factors for the variation in degradation rates. The typical degradation compounds observed for an aqueous MEA solvent were also observed for MEA in various concentrations and with various organic diluents.

## 1 Introduction

Greenhouse gas control is a key factor in reducing climate change. For postcombustion carbon capture, chemical absorption of carbon dioxide in aqueous amine solvents is a well-established technique and the current industry standard.^1^ Flue gas containing CO_2_ is brought into contact with an amine in an absorption column, with which it selectively reacts. Purified gas exits the system, while the separated CO_2_ is released from the amine upon heating in a desorber column. Numerous amine systems have been experimentally investigated and maybe the most well-known systems are 30 wt % aqueous monoethanolamine (MEA) and 40 wt % piperazine (PZ)/amino-methyl-propanol (AMP).^2−4^

During the absorption/desorption process of carbon dioxide, the amines undergo unwanted irreversible reactions. This is due to the harsh environment they are exposed to in the cyclic system, such as exposure to reactive components in the flue gas, elevated temperatures, and contact with metals.^5,6^ This amine degradation causes a significant operating expense and is one of the key issues with this technology. The compounds formed during degradation of the amines cause foaming, increased viscosity, corrosion of equipment, and fouling.^7,8^ Also, emissions of hazardous degradation compounds and makeup cost for treatment of the old solvent are challenges for the process.^6,9,10^ Therefore, reducing degradation is essential to make this technology acceptable for large emission industries, such as waste incineration, cement and steel production, and fossil-fuel-based energy production.^11^

To achieve this reduction, understanding the process behind the degradation is essential. Having an improved understanding of the underlying chemistry can help improve existing solvent systems, and, in addition, help in the development of new solvent systems. In this work, we will investigate the effect of solvent composition on thermal degradation. This has been done through a series of lab-scale experiments. First, we investigated how water in the solvent blends affected the degradation. This was done through thermal degradation experiments of various amine blends. Water was removed by changing the concentration of the amine. Thereafter, water was removed by switching the water with organic diluents. In this way, we could study the effect of both water and organic diluents on the thermal stability of amines. MEA was chosen as a reference system, as it is an already well-studied amine, and other amines were included based on their structure.

### 1.1 CO_2_ Absorption

**Scheme 1:**
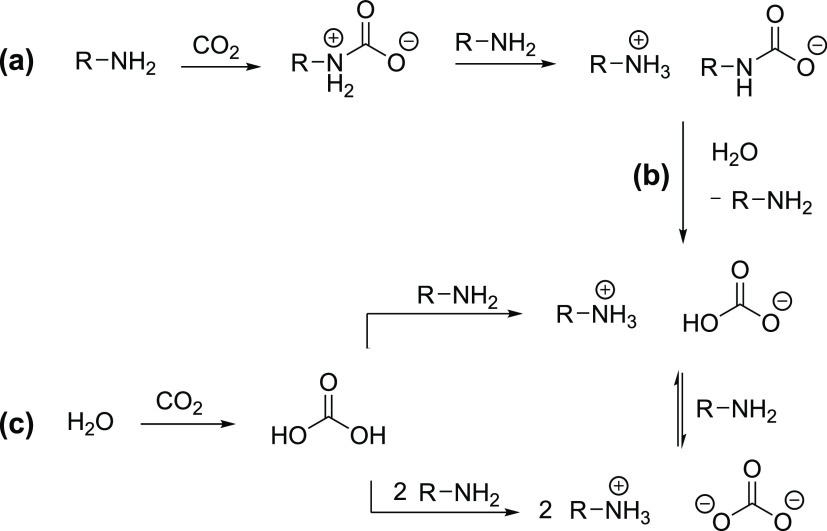
CO_2_ Absorption Pathways for Primary Aqueous Amines

The chemical absorption of CO_2_ is an acid–base reaction between carbon dioxide and the amine absorbent. Scheme 1 shows an overview of the reaction pathways for the formation of CO_2_-carrying species upon loading an aqueous primary amine solution. For primary and secondary amines, the acidic CO_2_ can react with two moles of amine forming an amine carbamate, possibly via a zwitterion, as shown in Scheme 1a.^12,13^ The lifetime of this zwitterion is uncertain, but it is expected to be unstable.^14,15^ Da Silva and Svendsen^16^ found that the carbamate formation is likely to happen through a one-step reaction, where the zwitterion is entirely transient, or, if the zwitterion is formed as an intermediate, is likely to be short-lived. Tertiary amines cannot undergo this reaction to form carbamates as their three substituents make them unable to transition from the zwitterion to a stable carbamate.

The reaction between primary, secondary, or tertiary amines and CO_2_ can also lead to the formation of bicarbonate and carbonate salts if water is present in the system. This can happen by hydrolysis from the amine carbamate, as shown in Scheme 1b, or through the formation and deprotonation of carbonic acid, as shown in Scheme 1c. This last case is the main route for tertiary amines reacting with CO_2_. Generally, when an amine reacts with CO_2_, protonated amines are formed as the counter ions to the CO_2_-carrying products.^17,18^ Physical absorption also occurs and is favored at high CO_2_ pressures.

Which CO_2_-carrying species are formed is governed by the conditions of the system, e.g., choice of amine, amine concentration, pH of the solution, etc. A primary amine, such as MEA in an aqueous solution, would, for example, primarily form MEA carbamate.^17^ The formation of carbonate, even though experimentally observed, is not considered significant for CO_2_ absorption in aqueous MEA.^19^

### 1.2 Thermal Degradation Mechanism

Degradation of the amines is generally categorized as oxidative or thermal, either with or without the presence of CO_2_. Little degradation has, however, been observed in aqueous amine solutions without CO_2_, even at 200 °C.^20^ Thermal degradation in the presence of CO_2_ is dependent on temperature and therefore happens mainly in the stripper and reboiler. It has been studied experimentally for a long time,^21^ and a polymerization reaction has been proposed.^22,23^ The reaction rate has been found to increase with higher temperatures, pressures, and higher concentrations of CO_2_.^5,24,25^

**Scheme 2:**
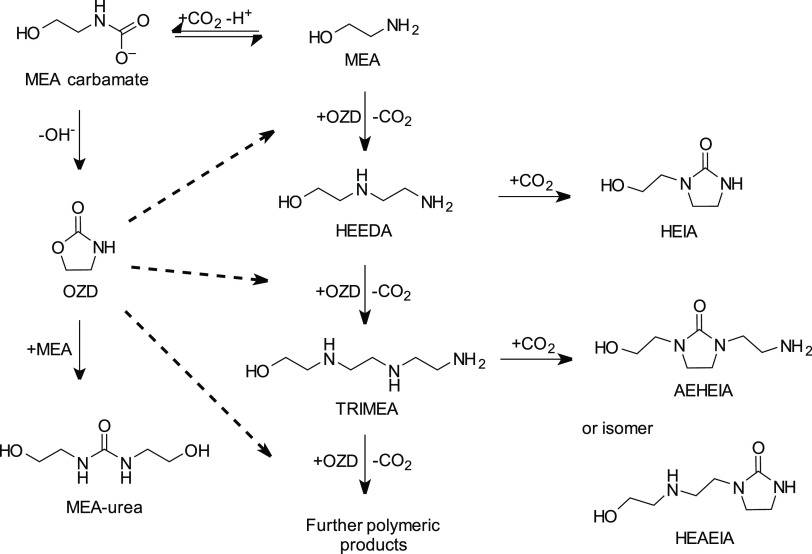
Overview of the Proposed Carbamate Polymerization Reaction of MEA under Stripper Conditions^27^

Scheme 2 shows an example of the polymerization reaction for thermal degradation of MEA. When MEA reacts with CO_2_, MEA carbamate is formed. The carbamate polymerization reaction is thought to be initiated by intramolecular cyclization of this carbamate or its protonated form, carbamic acid. The cyclization reaction results in the formation of 2-oxazolidone (OZD).^24,26^ This reactive degradation compound is found only in small concentrations and is thought to be an intermediate product, reacting with MEA to form other identified degradation products, such as *N*-(2-hydroxyethyl)-ethylenediamine (HEEDA/AEEA), 1-(2-hydroxyethyl)-2-imidazolidone (HEIA), 1,3-bis(2-hydroxyethyl)urea (MEA urea/BHEU), *N*-(2-hydroxyethyl)-diethylenetriamine (TRIMEA), etc. The order and by which mechanisms these are formed is not fully established, and different scenarios have been proposed.

The formation of OZD is believed to institute further amine degradation through the carbamate polymerization reaction. Initially, Polderman et al.^22^ proposed that HEIA is formed when OZD reacts with MEA. From this, HEEDA was believed to form when HEIA expelled a CO_2_ molecule. Davis^23^ has later proposed that HEEDA is actually formed from OZD reacting with MEA. From experiments performed under stripper conditions, it was found that very little HEEDA is formed from HEIA, whereas HEIA is readily formed from HEEDA when exposed to CO_2_. Degradation experiments have shown that, after an initial increase, the concentration of HEEDA has been found to stabilize, indicating that it acts as an intermediate. HEIA has been found to accumulate over time.^28,29^ This supports the proposed mechanism by Davis.

Similar to HEEDA, MEA urea can also be formed from MEA reacting with OZD. Which of these degradation product, HEEDA or MEA urea, is formed depends on where on the OZD structure the ring is cleaved. The formation of HEEDA expels one CO_2_ molecule, while for the formation of MEA urea, it is kept intact.

Further polymeric degradation products can be formed from HEEDA and OZD. Davis^23^ found that HEEDA can react with OZD to form TRIMEA, likely following the same reaction mechanism as that of the formation of HEEDA itself. TRIMEA can then either react with another OZD, following the same reaction mechanism, to form further polymeric products or with CO_2_ to form cyclic urea. This intramolecular ring closure gives rise to two possible degradation products, HEAEIA and AEHEIA. Since they are constitutional isomers and standards for MS analysis have not been commercially available, it is unknown which isomer is formed.

Tertiary amines do not degrade through the same reactions, simply because they do not form carbamate upon CO_2_ loading. These need a preliminary step of dealkylation to form a primary or secondary amine before further degradation can occur. Because of this, tertiary amines are considerably more thermally stable than primary and secondary amines.^28^

### 1.3 Water-Lean Solvents

Water-lean, hybrid, or mixed solvents are common denominators of solvent systems, where the water content is reduced. In recent years, water-lean solvents have been proposed in the literature as an option to reduce the energy consumption for solvent regeneration.^30,31^ In utilizing these solvents, the objective is to keep the high efficiency of aqueous alkanolamines but reduce unwanted properties.^32^ Amongst others, this includes the high energy cost of the vaporization of the cosolvent, which for aqueous amine systems is the water.

The term water-lean solvent covers a broad array of solvents. In some cases, the amine concentration in aqueous amine blends is simply increased, thereby replacing the water with an amine. Some proposed solvents have water replaced in parts or in total by an organic diluent. In other cases, chemical classes with more complex binding mechanisms are proposed. In total, a considerable amount of solvent mixtures have been tested, ranging from blends with the typical organic cosolvents to the more advanced CO_2_ binding organic liquids, known as CO_2_BOLs.^19,32−40^ Among these, there are some very promising solvents being tested at a pilot scale, such as RTI international’s NAS, ION’s advanced solvent, GE Global Research’s GAP-TEG solvent system, and PNNL’s EEMPA solvent.^41−44^ These are examples of water-lean solvents systems where the positive traits of water-lean solvents have been maintained, while lower degradation compared to the standard aqueous MEA has been achieved. Thus, these solvents are providing a possible interesting future for CO_2_ capture. Still, even though many water-lean solvent systems have been proposed and studied, there is still little data published on how the changes in composition of the solvent influence the degradation of the amines.^30^

## 2 Materials and Methods

### 2.1 Experimental Procedure

The chemicals used for this study are listed in Table 1.

**Table 1:**
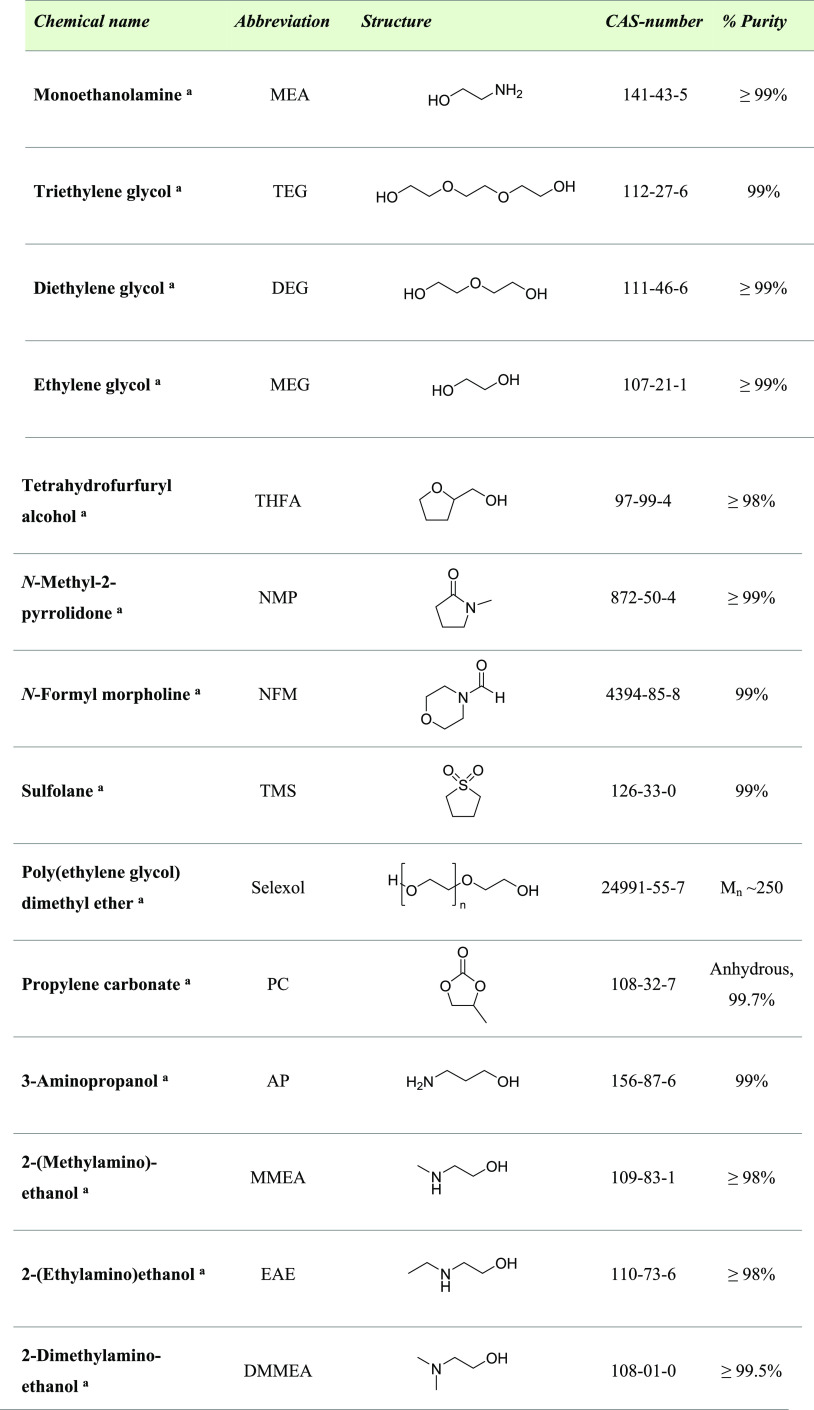

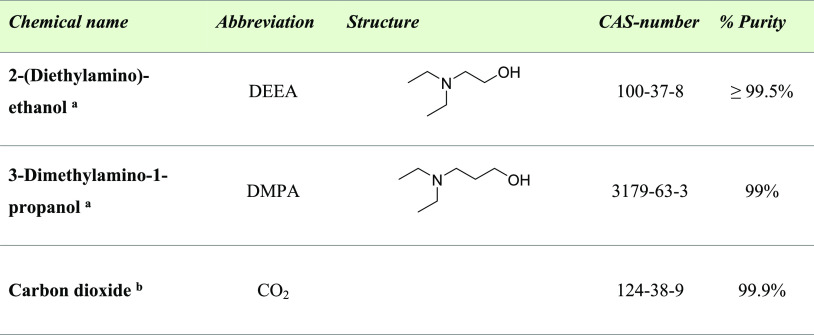
Details on the Chemicals Used in This Study

a Purchased from Sigma-Aldrich Norway AS/Merck Life Sciences.

b Purchased from AGA AB.

The effect of solvent on the thermal stability of amines was studied in the presence of CO_2_. This has been done through four series of experiments. An overview of the experiments is given in Table 2.

**Table 2:** Overview of Thermal Degradation Experiments

Amine	Amine concentration	Loading [mol CO_2_/mol amine]	Organic solvent	Organic solvent concentration [mol %]
**Variations of Loaded MEA**				
MEA	11 mol %	0.1, 0.2, 0.3, 0.4		
	8–100 mol %	0.1–0.5^a^		
	11–73 mol %	0.1		
**MEA in TEG and Water**				
MEA	5	0.5	TEG	0-100
**Various Amines in TEG and Water**				
AP, MMEA, EAE	5	0.5	TEG	0, 50
DMMEA, DEEA, DMPA		0.3		
**MEA in Various Organic Solvents**				
MEA	43 mol %	0.5	MEG, DEG, TEG, THFA, NMP	100
			NFM	20

a The loadings were chosen to obtain a constant absolute amount of CO_2_ (0.19 mol CO_2_ per 100 g unloaded solution) with the varying MEA concentration.

#### Variations in MEA and CO_2_ Concentrations

Solutions with different ratios of MEA and deionized water were prepared. Three different sets of solutions were made by changing different parameters: (1) Solutions where the MEA concentration was kept constant (11 mol %, 7 ) and the loading was varied, (2) solutions where the MEA concentrations were varied, and the absolute CO_2_ concentration was kept constant (0.19 mol CO_2_ per 100 g unloaded solution), and (3) solutions where the MEA concentrations were varied while the loading was kept constant (0.1 mol CO_2_ per mol MEA).

#### MEA in Varying Ratios of Triethylene Glycol (TEG) and Water

Solutions of 5  MEA in varying ratios of TEG and deionized water were prepared. The ratios of TEG and water ranged from 0–100%. All of the solutions were loaded to 0.5 mol CO_2_ per mol MEA.

#### Other Amines in TEG and Water

Various amines were prepared in solutions (5 ) with both pure deionized water and with 50 mol % TEG in water. The amines studied were the primary amines MEA and AP, the secondary amines MMEA and EAE, and the tertiary amines DMMEA, DEEA, and DMPA. Solutions of primary and secondary amines were loaded to 0.5 mol CO_2_ per mol amine, while solutions of tertiary amines were loaded to 0.3 mol CO_2_ per mol amine. The loading of 0.3 for the tertiary amines was chosen because it was not possible to reach loadings of 0.5 in solutions containing TEG.

#### MEA in Various Organic Solvents

Solutions of 43 mol % MEA in various organic solvents were prepared. MEA (43 mol %) was chosen as this corresponds with the MEA concentration of the solution already studied for MEA in pure TEG. A wide array of diluents proposed as candidates for water-lean applications was tested.^30^ The organic solvents chosen were monoethylene glycol (MEG), diethylene glycol (DEG), *N*-methyl-2-pyrrolidone (NMP), tetrahydrofurfuryl alcohol (THFA), *N*-formylmorpholine (NFM), sulfolane (TMS), poly(ethylene glycol) dimethyl ether (Selexol), and propylene carbonate (PC). However, both TMS and Selexol formed two phases upon loading, and MEA in PC could not be loaded above 0.1 mol CO_2_ per mol MEA. MEA in pure NFM also resulted in phase separation, but here, the addition of water gave one phase. Therefore, a sample of MEA in 20 mol % NFM in water was run. In conclusion, the solvents that were tested in thermal degradation experiments with MEA were monethylene glycol (MEG), diethylene glycol (DEG), triethylene glycol (TEG), tetrahydrofurfuryl alcohol (THFA), and *N*-methyl-2-pyrrolidone (NMP), as well as a mixture of 20 mol % *N*-formylmorpholine (NFM) in water. All solutions were loaded to 0.5 mol CO_2_ per mol MEA.

All solutions were made and loaded gravimetrically in batches, and loading was achieved by sparging CO_2_ gas into the solutions. Both loading and amine concentration were checked with amine titration and total inorganic carbon (TIC) analysis, respectively. All solutions were initially slightly overloaded to allow correction of the concentrations by adding the fresh solvent to the batch before the experiments were run.

Thermal degradation of the solutions was conducted in 10 cm long 316 stainless steel cylinders, with an outer diameter of 1.3 cm and thickness of 0.1 cm, and equipped with Swagelok end caps. For each solution, the same batch was loaded into a set of 10 cylinders, giving five data points over time and two parallels. The average relative standard deviation between the parallels was 0.49%. Within this, there were three parallels with a distinctly higher relative standard deviation of 2.0–3.5%. These are given in Supporting Information Table S1. For all series, the solution (8 mL) was loaded directly into the cylinders. The cylinders were closely sealed and placed in a forced convection oven at 135 °C. This temperature was chosen as it is the temperature frequently reported in other studies, which allows for comparison of degradation data with these publications.^24,26,45−48^ For all series with primary and secondary amines, cylinders were extracted once a week, while for the series of tertiary amines, cylinders were extracted over a longer period. This was to ensure enough degradation from the more stable tertiary amines.

Metal cylinders opened for sampling were not returned for further degradation. All cylinders were weighed before and after the experiment to detect possible leakages. Leakages were detected in 7 of the 275 cylinders. An overview of the cylinder leakages is given in Supporting Information Table S2. For these solutions, the results reported are from only one parallel.

A selection of the solvent blends was also introduced into glass tubes (4 mL), which were then placed into new sets of cylinders. The glass tubes were used to prevent contact between the solvent and the metal walls of the cylinders. There was no significant difference in degradation rate for the experiments done with the solutions in direct contact with the cylinder compared to the ones with inserted glass walls—see comparison in Supporting Information Figure S1. Quantitatively and qualitatively, the formed degradation products were also the same in both cases—see comparison in Supporting Information Figure S2. This indicates that the degradation mechanisms are not influenced by the metal concentration in the solutions. This is in line with the literature.^49^

### 2.2 Analytical Methods

Total alkalinity of the samples was found through amine titration with H_2_SO_4_.^50^ Total inorganic carbon (TIC) measurements were used to determine the amount of CO_2_ in the samples. For this, a Shimadzu TOC-L_CPH_ in TIC mode was used. Combining TIC results with the titration allowed us to monitor the loading of the solutions.

Quantitative analyses of MEA and a selection of thermal degradation products by Liquid chromatography coupled with mass spectroscopy (LC-MSMS) were performed by SINTEF Industry on a UHPLC Agilent 1290 Infinity System with an Agilent 6490 triple quadrupole detector. For analyte separation, both Ascentis Express Phenyl-Hexyl 2.7 μm HPLC column and a Discovery HS F5 HPLC column from Sigma-Aldrich Co. LLC were used.

The NMR experiments were performed at 26.8 °C on a Bruker 600 MHz Avance III HD equipped with a 5 mm cryogenic CP-TCI z-gradient probe. The obtained spectra were analyzed in the software Bruker TopSpin 4.0.7. Deuterated water was used as the “lock” solvent and TMSP (Tris(trimethylsilyl)phosphine) was used as an internal reference standard. The solution to be analyzed was placed in an NMR tube, and the “lock” solvent was placed in an inserted coaxial insert.

## 3 Results and Discussion

Our focus in this paper is to investigate what effect water and the solvent composition have on thermal degradation of amines. This has been done through four series of thermal degradation experiments. Results from the variations of loaded aqueous MEA will be presented in Section 3.1. In Section 3.2, results from both the series of MEA in different ratios of TEG and water and the series of various other amines in TEG and water will be presented. The last of the series will be presented in Section 3.3 and covers thermal degradation results of MEA in various organic solvents. The thermal degradation products found in the different series will be presented in Section 3.4.

The degradation trends of the solvent amine presented in this section will be derived from titration results. The titration results show the solvent’s basicity, and since some degradation products are basic, the titration results will somewhat overpredict the actual concentration of the starting amine (e.g., MEA). However, even though titration measurements are not as accurate as other analytical methods, such as the LC-MS analysis, more data points are available due to the simplicity of the method. As the trends coincide well with the more accurate LC-MS results, titration data will be used when presenting the degradation trends. See the Supporting Information, Figure S3, for a comparison of titration and LC-MS data. All thermal degradation data presented in this section will be given as figures. Data for these and associated analyses will be provided in Supporting Information Tables S3–S11.

### 3.1 Variations of Loaded MEA

In the first series of experiments, our focus was on studying the effect of solvent composition in aqueous amine solutions. To achieve this, three variations on loaded aqueous MEA were studied. The three groups were solutions, where (1) the MEA concentration was kept constant and the loading was varied, (2) the MEA concentration was varied and the absolute CO_2_ concentration was kept constant, and (3) the MEA concentration was varied while the loading was kept constant.

**Figure 1:**
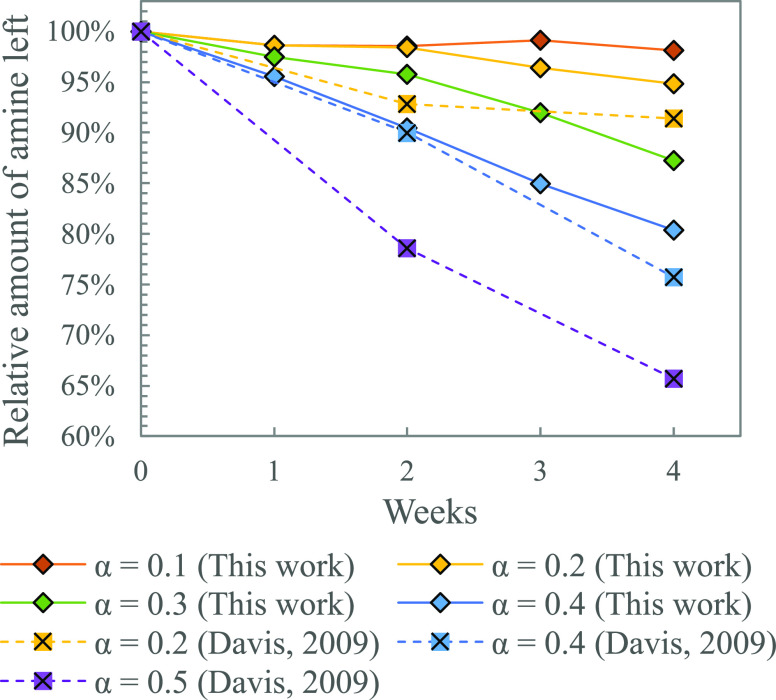
Effect of CO_2_ loading (α) on thermal degradation of MEA (11 mol %, 135 °C). Data points from Davis and Rochelle^24^ represent purely MEA left in the solutions as LC-MS was used for analysis.

The first series of aqueous MEA were solutions of 11 mol % MEA (30 wt %) with increasing loadings (α = 0.1, 0.2, 0.3, and 0.4). Figure 1 shows the titration results from this experiment, plotted together with LC-MS data from thermal degradation experiments performed under the same conditions by Davis and Rochelle.^24^ Da Silva et al.^45^ have also reported data showing the same trends but with distinctly higher degradation rates. The results show that thermal degradation increases with increased loading in all cases.

**Figure 2:**
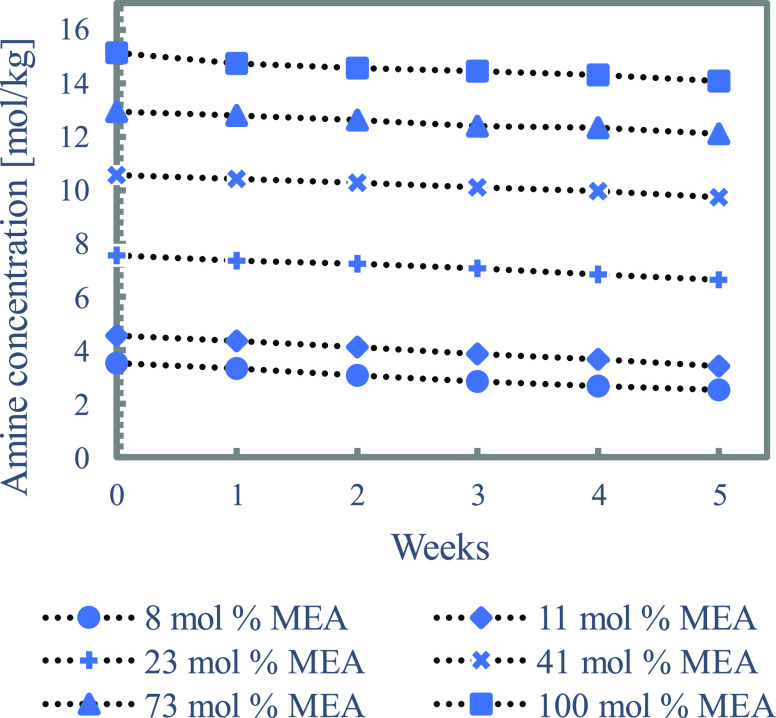
Effect of MEA start concentration on thermal degradation of loaded MEA (0.19 mol CO_2_/100 g unloaded solution, 135°C).

The second series of solutions consisted of increasing ratios of MEA in deuterated water, ranging from 8–100 mol % MEA before loading. All solutions were loaded with 0.19 mol CO_2_ per 100 g unloaded solution, corresponding to the amount needed to load the 8 mol % solution to 0.5 mol CO_2_ per mol MEA. This means that while the loading decreases with the increase in MEA concentration, the concentration of CO_2_ is the same for all solutions. Figure 2 shows the resulting amine loss and indicates that all solutions degrade at approximately the same rate. The absolute amine loss for all of the solutions lies within 0.81–1.14 mol/kg and is not following any trend. It, therefore, appears that the thermal degradation rates are closely tied to the absolute amount of CO_2_. This is in line with literature findings.^51^ From these results, and those from the previous experiment, it also appears that the thermal degradation rates do not seem to be affected much by MEA or water concentration.

**Figure 3:**
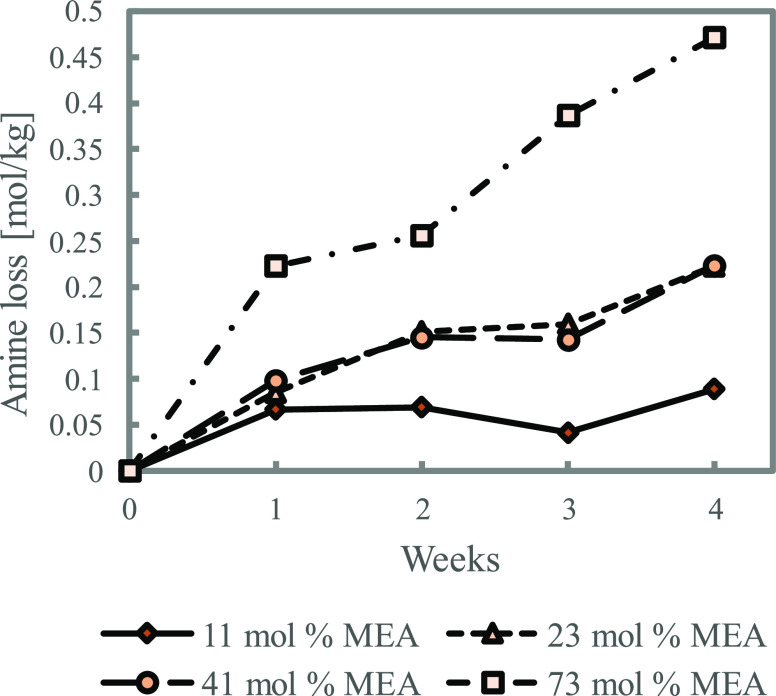
Effect of change in amine concentration on thermal degradation of loaded MEA (α = 0.1, 135 °C).

The third series of aqueous loaded MEA was solutions of increasing amounts of MEA with loadings of 0.1. This loading was chosen to avoid high pressures in the cylinders at high MEA concentrations, unfortunately at the expense of higher degradation rates. The results shown in Figure 3 are as expected. With higher MEA concentrations and, therefore, higher CO_2_ concentrations, the amine loss was bigger.

From these three experiments, it seems like mainly the CO_2_ concentration is accountable for the rate of thermal degradation. However, the change in loading should not necessarily be seen simply as changes in CO_2_ concentration in the different solutions. As presented in Section 1.1, changes in the loading and/or solvent composition will change which CO_2_-carrying species are being formed and in what quantities. Therefore, we plotted the equilibrium speciation of loaded MEA solutions for different loadings and MEA concentrations using the ENRTL model in Aspen Plus V10.

**Figure 4:**
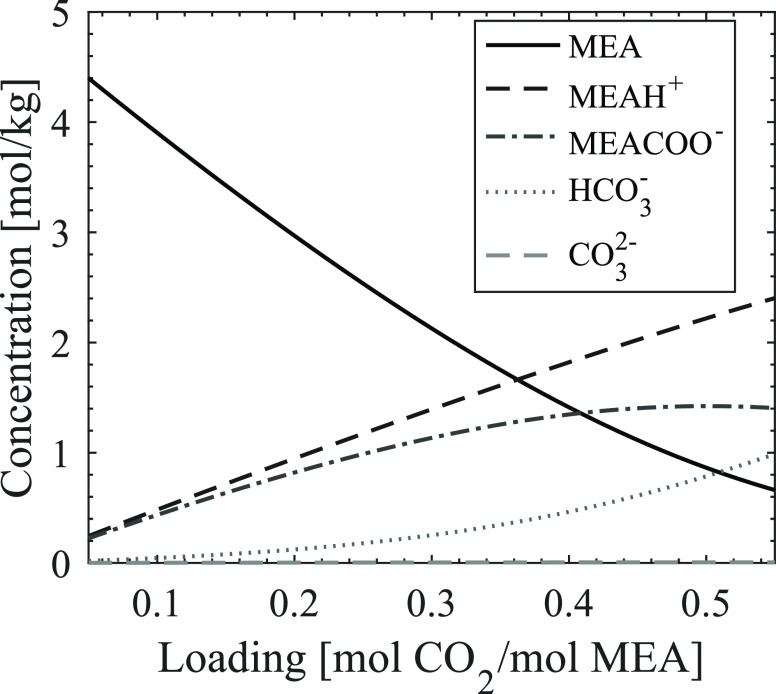
Speciation upon loading of 11 mol % aqueous MEA (speciation given by the ENRTL-RK model in Aspen Plus V10, 135 °C).

We start by looking at the solutions with increasing loading and constant MEA concentration. Figure 4 shows the predicted speciation for different loadings of 11 mol % aqueous MEA at 135 °C. As expected for a primary aqueous amine, MEA carbamate is the predominant CO_2_-carrying species formed upon loading. At low loadings, carbamate is almost exclusively forming, while as the loading increases, some fractions of CO_2_ in the solutions form bicarbonate. Protonated MEA is formed as the counterion for both carbamate and bicarbonate and therefore increases steadily with the increase in loading.

MEA carbamate is thought to play a prominent role in the initial carbamate polymerization reaction.^20^ In Figure 1, it was seen from the experiment that increased loading resulted in increased thermal degradation. The degradation rate for the solution loaded to 0.5 is distinctly higher than that for the solution loaded to 0.4. At the same time, Figure 4 shows that the MEA carbamate concentration at loadings 0.4 and 0.5 is quite similar, indicating that the MEA carbamate concentration alone is not accountable for the reaction rate during thermal degradation. The degradation rates correlate to the total amount of CO_2_ present, seemingly regardless of in which form, and thereby also to the concentration of protonated MEA. Regarding identifying a rate-limiting component, both total CO_2_ concentration and protonated MEA concentration are viable possibilities.

**Figure 5:**
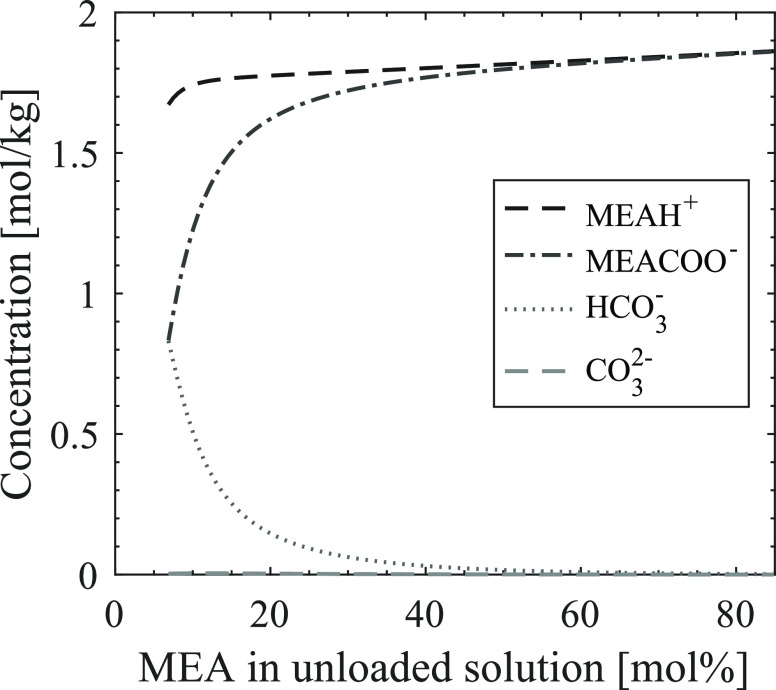
Speciation at increasing concentrations of MEA (speciation given by the ENRTL-RK model in Aspen Plus V10, 135 °C, [CO_2_] = 1,9 mol/kg).

Next, we look at the speciation of the solutions with increasing MEA concentration and constant absolute CO_2_ concentration. Figure 5 shows the predicted speciation of the CO_2_-carrying species in these solution compositions at 135 °C. Here, we see that at higher mole percentages of MEA, mainly the formation of carbamate and protonated MEA is expected. At the lower percentages, i.e., with the presence of more water, increasing amounts of bicarbonate take the place of carbamate. The thermal degradation results of these solutions, as presented in Figure 2, showed that the solutions with low MEA concentrations (e.g., 8 mol %) degrade at the same rate as solutions with high MEA concentrations (e.g., 73 mol %). Seeing these results in context with the predicted speciation of the system, the concentration of carbamate and bicarbonate is predicted to be approximately the same at 8 mol % MEA, while it is predicted to only form carbamate at 73 mol % MEA. Again, the MEA carbamate concentration alone does not seem to influence the degradation. Also, both the concentration of protonated MEA and the total concentration of CO_2_-carrying species correlate better with the observed degradation rates. In the end, the correlations found from these results do not allow us to determine one species that governs the degradation rates. However, investigation of the impact of these species could be an interesting aspect for future work.

From these thermal degradation results of aqueous MEA solutions, it seems like the concentration of CO_2_ in the solutions affects the degradation rates, while the concentration of water does not. To investigate this further, we then looked more closely for any effect of removing water. In the previous experiments, this was done by changing the ratios of MEA and water. Now, we wanted to look at the effect of replacing water with an organic solvent.

### 3.2 MEA and Other Amines in Various Ratios of TEG and Water

While switching water to an organic diluent allowed us to study the effect of the water, it also gave an insight into the stability of amines in nonaqueous and water-lean systems. Triethylene glycol (TEG) was chosen as the organic diluent in the first experiments because it was expected to be inert and stable under the given conditions. The effect of removing water was studied by comparing results for an aqueous solution with results for solutions, where increasing amounts of water were replaced with TEG.

**Figure 6:**
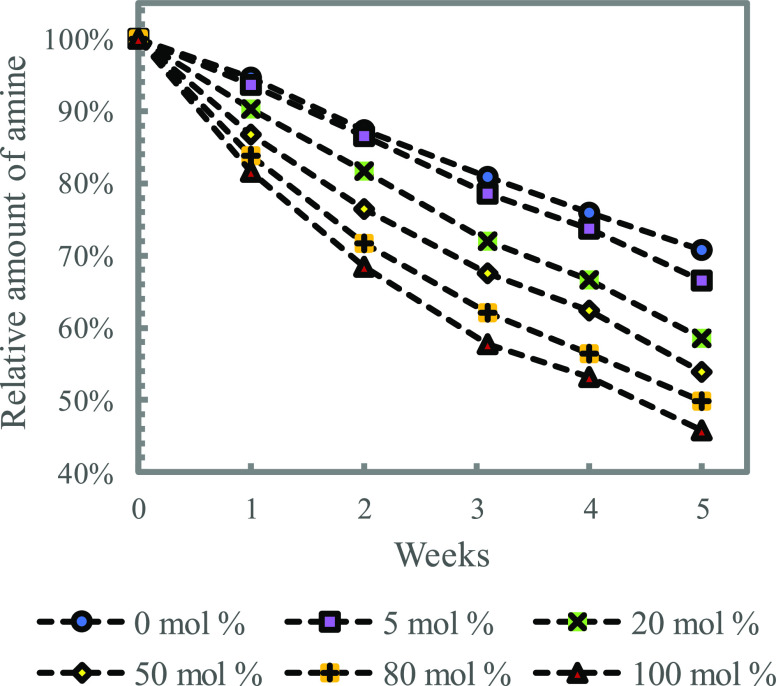
Effect of increasing ratios of TEG in water on thermal degradation of MEA (5 , α = 0.5, 135 °C).

Solvent blends with 5  MEA loaded to 0.5 mol CO_2_ per mol MEA were prepared in TEG and deuterated water solutions ranging from 0–100 mol % TEG. Figure 6 shows that higher ratios of TEG in the solvents resulted in increased thermal degradation rates of MEA.

Trying to explain the reduced stability in the organic diluent, we started by looking into the thermal stability of TEG itself. The idea behind this was that if TEG thermally degrades, compounds that are formed from this might enhance the degradation of MEA. This would explain the degradation rates observed. A thermal degradation experiment of pure TEG was therefore conducted with the same conditions that were used for the mixed solvent. After five weeks, a sample of the degraded TEG was analyzed using NMR spectroscopy. A comparison with a sample of pure, undegraded TEG showed that TEG had undergone virtually no thermal degradation. This explanation can then be ruled out. The comparison of the two NMR spectra is given in Supporting Information Figure S4.

Looking into other possibilities for the increase in degradation with increased ratios of TEG, we then considered if the presence of TEG leads to the emergence of other degradation pathways. An indicator of this might be the presence of other degradation products than the ones expected through the carbamate polymerization reaction of aqueous MEA. We, therefore, analyzed for these degradation products to see if they would account for the amount of MEA lost during the degradation. Using LC-MS to search for the known thermal degradation products of MEA, 2-oxazolidone (OZD, CAS: 497-25-6), 1,3-bis(2-hydroxyethyl)urea (MEA urea, CAS: 15438–70–7), *N*-(2-hydroxyethyl)ethylenediamine (HEEDA, CAS: 111-41-1), and 3-(2-hydroxyethyl)-1,3-oxazolidin-2-one (HEIA, CAS: 3356-88-5) were observed. Results can be found in Section 3.4. The formation of further polymerized degradation products, e.g., tri-HEIA, was not explored, as an analytical method for this was not available. Moles of unknown degradation products, in relation to the amine loss in the solutions, were slightly higher in the solutions with TEG than the ones from the aqueous solution (8 mol % MEA). Thus, these results do not clearly indicate that other degradation pathways are present. It is, however, clear that aqueous MEA solutions and MEA solutions with organic solvents, such as TEG, form many of the same degradation compounds. More details on the thermal degradation products formed in these experiments will be presented in Section 3.4.

In the case of the increasing degradation rates for solutions with TEG, we will lastly touch shortly upon two other possible explanations: first, the ratios between the CO_2_-carrying species can be affected by the solvent change and, second, the solvent properties of the organic diluent have other effects on the species involved in the degradation mechanism.

As already stated in earlier discussions, the main CO_2_-carrying products in aqueous MEA loaded to 0.5 are MEA carbamate and bicarbonate, both paired with protonated MEA. This is what we expect to be formed with the presence of water. Bicarbonate needs the presence of water to be formed. Removing water from the system, therefore, changes the speciation of the loaded solutions. Also, TEG is known to form alkylcarbonate in the presence of CO_2_.^52^ These are, however, not expected to form in significant amounts if carbamate can be formed. Unfortunately, we do not have a model for the speciation under these conditions to investigate this further.

The other effect of changing the solvent might be how well it stabilizes the different compounds involved in the carbamate polymerization reaction. Water is highly polar and is an excellent solvent for stabilizing ions. When switching to TEG, the ionic species, carbamate, and protonated MEA become less stabilized. The equilibrium between the ionic form and nonionic form of carbamate and protonated MEA might then be shifted toward the nonionic form. This results in higher concentrations of carbamic acid. If this protonation step is necessary for the reaction to form OZD, then this might be an explanation to why degradation rates are enhanced. Overall, it becomes clear from this that further studies are needed to explain the change in stability.

**Figure 7:**
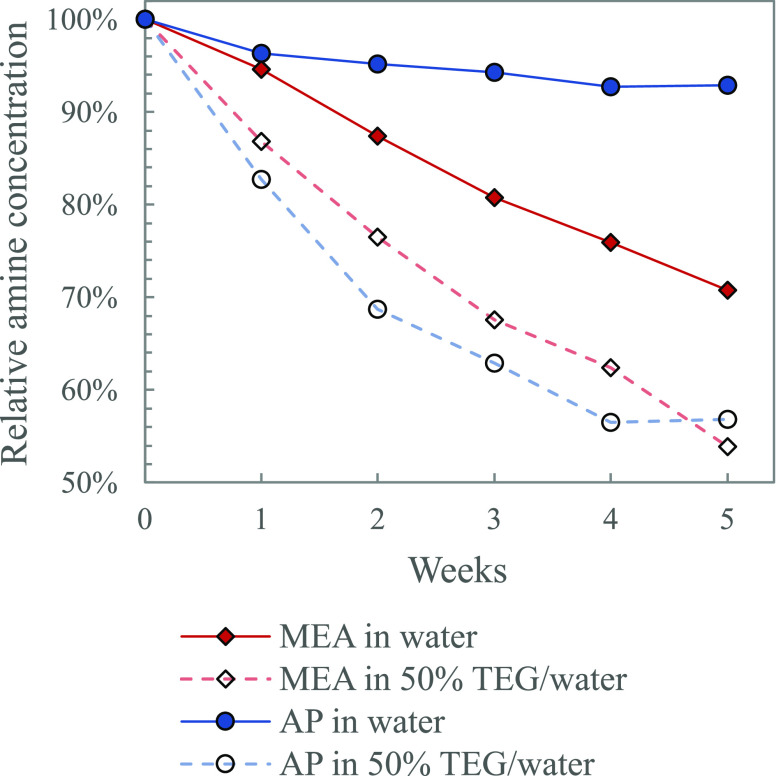
Effect of addition of TEG on the thermal stability of primary amines MEA and AP (5 , α = 0.5, 135 °C).

**Figure 8:**
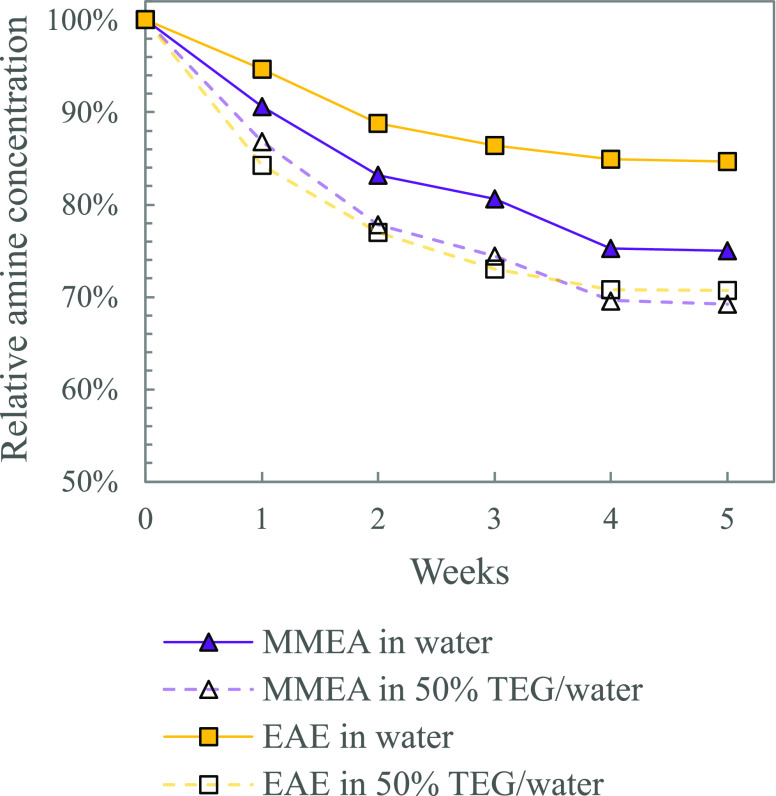
Effect of addition of TEG on the thermal stability of secondary amines MMEA and EAE (5 , α = 0.5, 135 °C).

We also tested the thermal stability of some primary, secondary, and tertiary amines in solutions with 50 mol % TEG in water. As presented in Section 1.2, primary and secondary amines that form carbamate are believed to degrade through the same mechanism as MEA. From the thermal degradation study done for AP, MMEA, and EAE in TEG (5, α = 0.5), shown in Figure 7 and Figure 8, we saw loss in stability when water was replaced with TEG. This indicates that the trends seen for MEA are also expected for other primary and secondary amines. Tertiary amines do not form carbamate and therefore have a high thermal stability. Thermal degradation of DMMEA, DEEA, and DMPA in TEG (5 , α = 0.3) did not show any noticeable effect on the stability compared to aqueous solutions. The results from the thermal degradation experiments of the tertiary amines are presented in Supporting Information Figures S5 and S7.

### 3.3 MEA in Various Organic Solvents

**Figure 9:**
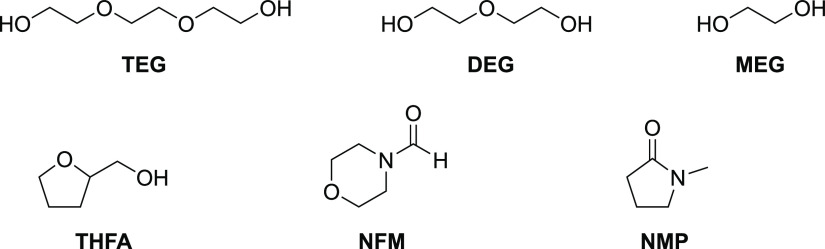
Structure of organic diluents.

**Figure 10:**
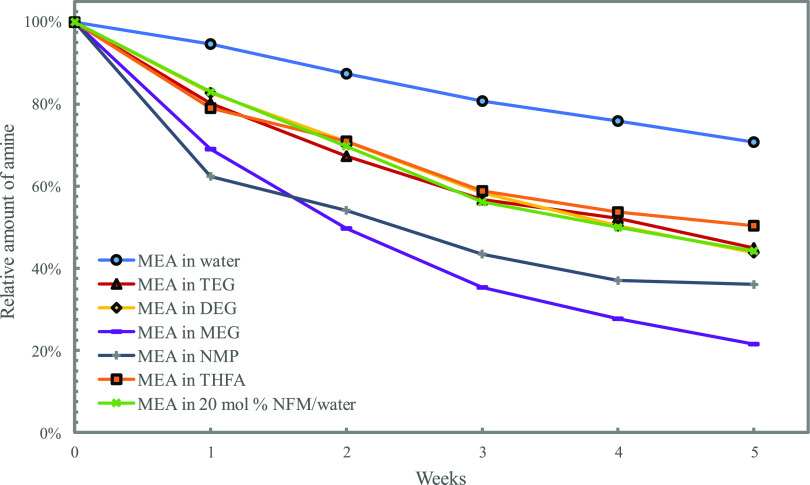
Overview of thermal degradation of loaded MEA in various organic diluents.

Seeing how the degradation of the amines increased when using TEG as the diluent, we then expanded our study of the effect of the solvent on thermal degradation by including other organic diluents. In this series of experiments, solutions of 43 mol % MEA in different organic diluents (DEG, MEG, THFA, NFM/water, and NMP, shown in Figure 9) were prepared and loaded to 0.5 mol CO_2_ per mol MEA. The degradation trends for all of the tested solutions are shown in Figure 10. All of the organic diluents increased the degradation rates of MEA compared to pure water.

The same reasoning as to why MEA degrades more in TEG than in water might apply to the other organic solvents as well. The difference in the degradation rates between the different diluents, however, is harder to explain. The organic diluents were selected based on what solvents were proposed as possible water-lean diluents in the literature, not their chemical properties. From the chosen collection, however, it is still possible to investigate the possible effect of some parameters such as acid–base behavior, polarity, and relative permittivities.

The acid–base behavior of the solvents is described by their autoprotolysis constants, pKs, also called p*K*_auto_. The pKs denote a solvent’s ability to self-ionize. Small values indicate that the solvent can easily donate a proton, and vice-versa.^52,53^ We only managed to find the p*K*_s_ values of water, MEG, and NMP, as presented in Table 3. NMP is considered an aprotic solvent, as it does not hold a proton attached to a heteroatom. Autoionization is therefore quite disfavored, which is denoted by the exceptionally large p*K* value of this solvent. From the p*K* values found and the thermal degradation rates of MEA in these three solvents, there does not seem to be a correlation. Though water and MEA have similar p*K* values, the degradation rate of MEA in MEG is considerably higher than that of MEA in water. The degradation rate of MEA in NMP, however, is closer to that of MEA in MEG, even with their distinctly different p*K*s.

**Table 3:** Solvent Properties of Pure Solvents: Autoprotolysis Constant (p*K*_s_), Relative Polarity (*E*_T_^N^), and Dielectric Constant (ε_r_)

Solvent name	p*K*_s_ [− lg(*K*_s_/mol^2^ · L^–2^)]	*E*_T_^N^ [−]^a^	ε_r_ [−]^a^
water	14^a^	1	78.36
MEG	15.84^a^	0.79	37.70
DEG		0.713	31.69 (20 °C)
TEG		0.682	23.69 (20 °C)
NMP	≥24.2^b^	0.355	32.2
NFM			
THFA			

a Ref (53).

b Ref (54).

The polarity parameter was touched upon in the previous section, and as stated there, water is more polar than TEG. The relative polarity of the solvents is presented in Table 3. Though this could seem promising as a way of explaining the decreased stability of the amine in TEG, it falls short when including the other organic solvents, e.g., MEG. MEG has a higher polarity than TEG, so by that reasoning, MEA should be more stable in this solvent. As seen from Figure 10, however, MEA in MEG has the highest degradation rate of the solvents tested.

The relative permittivity of a solvent is given by its dielectric constant, ε_r_. This value represents the solvent’s ability to separate charges and orient its dipoles. It has been found to influence the ability a solvent has to stabilize charged species.^53,55−57^ The dielectric constants for the studied solvents are presented in Table 3. There is again a mismatch between the investigated parameter and the observed thermal degradation rates. Suppose that a high dielectric constant allows the solvent to stabilize the MEA carbamate, thereby disfavoring the ring formation of OZD (see Section 1.2). The high dielectric constant of water is in line with this. The problem with this explanation arises when taking the glycols into account. MEA has a higher dielectric constant than DEG and TEG. However, it results in the highest degradation rate of MEA of the solvents studied.

None of the highlighted parameters give a satisfactory explanation for the degradation trends observed. The explanation thus might be a combination of different effects and might be specific to each solvent. As a final note, one can speculate if the reason for why MEA degrades faster in organic diluents than in water is an effect of the initial degradation reaction, the cyclization reaction forming OZD (see Section 1.2). In this reaction, a water molecule is expelled. This means that the formation of OZD should be more prone to happen in systems with lower water content. As OZD is considered the starting intermediate for the carbamate polymerization reaction, this increased formation rate would naturally lead to increased degradation rates for the system. The differences between the different organic diluents, however, is not explained by this.

Even though this study cannot give any exact mechanistic reasons for the increased degradation of the tested alkanolamines when water is replaced with the selected diluents, it does show why the degradation of water-lean solvents should be assessed in the early stage of solvent development work. It should also be remembered that most of the data in this work is on MEA-based water-learn solvent systems. Varying the amine (including the use of secondary and tertiary amines) would allow development of water-lean solvent systems with significantly lower degradation compared to aqueous 30 wt % MEA.

### 3.4 Degradation Product Formation

Lastly, we will present the thermal degradation products in the solutions containing MEA. The degradation products included are OZD, HEEDA, HEIA, and MEA urea. The other possible degradation products formed during thermal degradation of MEA shown in Scheme 2 were not analyzed as an analytical method was not available.

**Figure 11:**
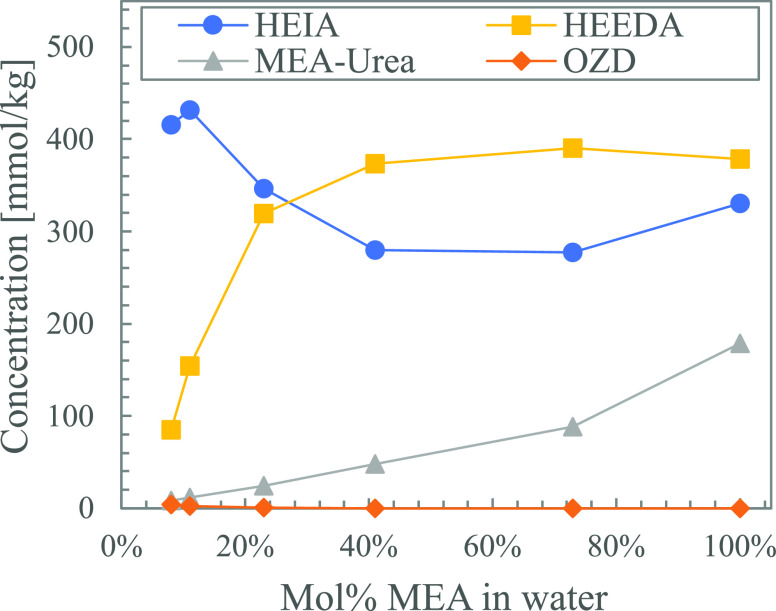
Thermal degradation products formed in solutions with increased concentrations of loaded MEA (0.19 mol CO_2_ per 100 g unloaded solution) after 5 weeks.

Figure 11 shows the thermal degradation products formed in solutions with varying ratios of loaded MEA in water (0.19 mol CO_2_ per 100 g unloaded solution) after five weeks. All solutions have the same absolute amount of CO_2_. The most drastic change when increasing the amine concentration is the steep increase in HEEDA. This can simply be because of the increasingly excessive amounts of MEA. OZD can thus readily react with MEA to form HEEDA. The high concentration of MEA also results in a shortage of free CO_2_ available. This can explain the decrease in HEIA, as it is expected to form through the cyclization of the carbamate of HEEDA. The amount of MEA urea formed also increases with increasing concentrations of MEA, which can be attributed to the ready availability of MEA.

**Figure 12:**
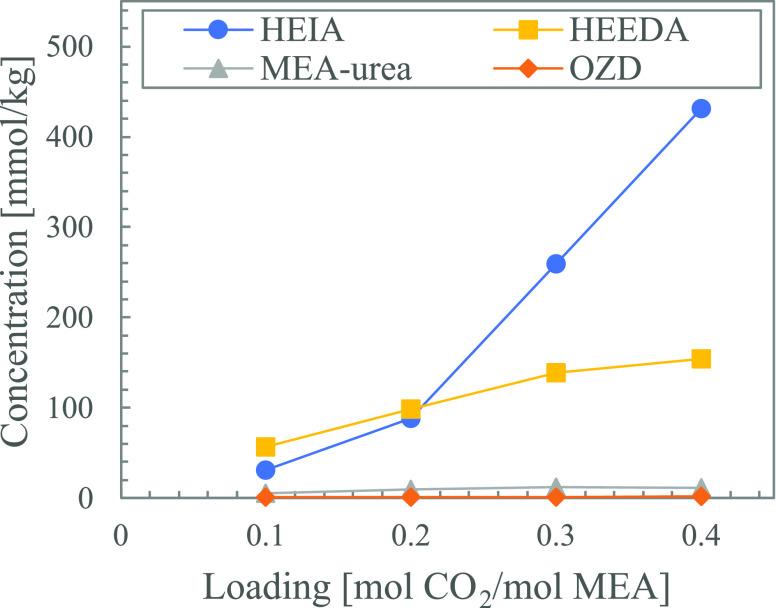
Thermal degradation products formed in solutions of loaded aqueous MEA (30 wt %) with increasing loading after 4 weeks.

Thermal degradation products formed after 4 weeks in aqueous solutions of MEA with different loadings are shown in Figure 12. The increase in loading resulted in a considerable increase in the formation of HEIA and a slight increase in the formation of HEEDA. HEIA formation is expected to be dependent on the amount of CO_2_ available to form the carbamate of HEEDA. The increase in HEEDA can therefore be seen as a result of the increase in CO_2_ concentration. This is in line with the results in Section 3.1, showing that increased loading resulted in increased thermal degradation.

**Figure 13:**
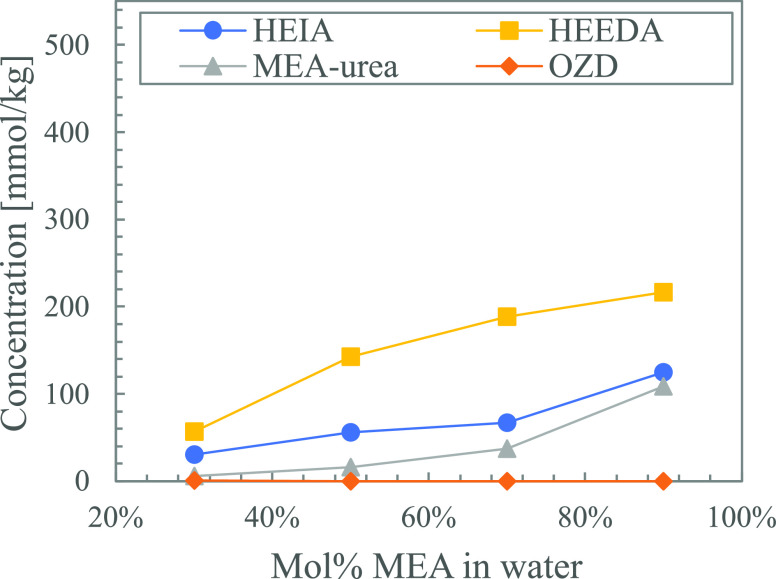
Thermal degradation products formed in solutions of loaded aqueous MEA (α = 0.1) after 4 weeks.

Aqueous MEA solutions with increasing amounts of MEA, all loaded to 0.1, gave thermal degradation products as presented in Figure 13. As the ratio between MEA and CO_2_ is kept constant, the results shown here are the effect of reducing the water content. The overall degradation rates (Figure 3) showed that the increased MEA concentration resulted in increased degradation. The increase in formation of HEIA, HEEDA, and MEA urea mirrors this. They all stay within the same trend, with only some small variations in the ratio between them. The OZD concentration stays low even with the increased MEA concentration. This is as expected since OZD is considered a short-lived intermediate product. Overall, the water concentration does not seem to influence the formation of thermal degradation products.

**Figure 14:**
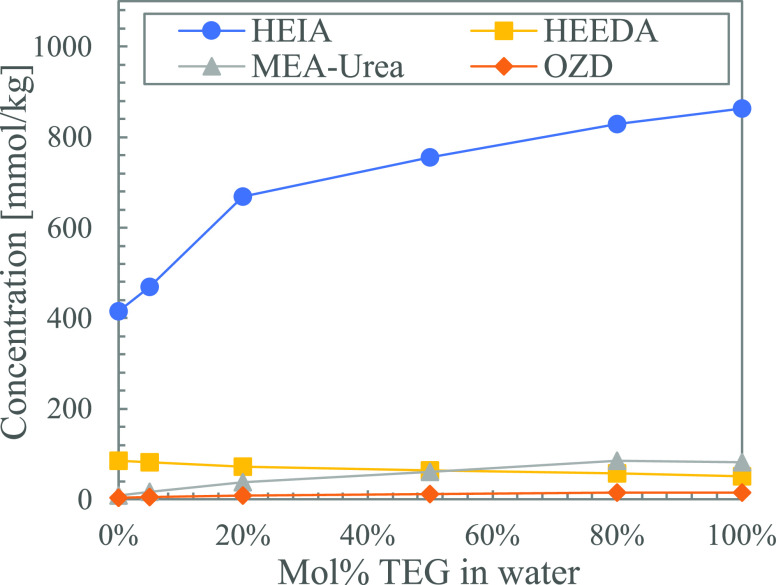
Thermal degradation products formed in solutions with loaded MEA (α = 0.5) in varying ratios of TEG and water after 5 weeks.

Figure 14 shows the thermal degradation products that are present in the solutions with loaded MEA in varying ratios of TEG and water at week 5. The data points at 0 mol % TEG correspond with the solution of 5  MEA (8 mol %) in water. Increasing the concentration of TEG, and thereby removing water, shows varying effects on the formation of thermal degradation products. HEEDA and MEA urea are only slightly affected and have a small decrease and increase, respectively. HEIA, however, is strongly influenced by the change in ratio. From solutions without TEG to solutions without water, the amount of HEIA produced is doubled.

**Figure 15:**
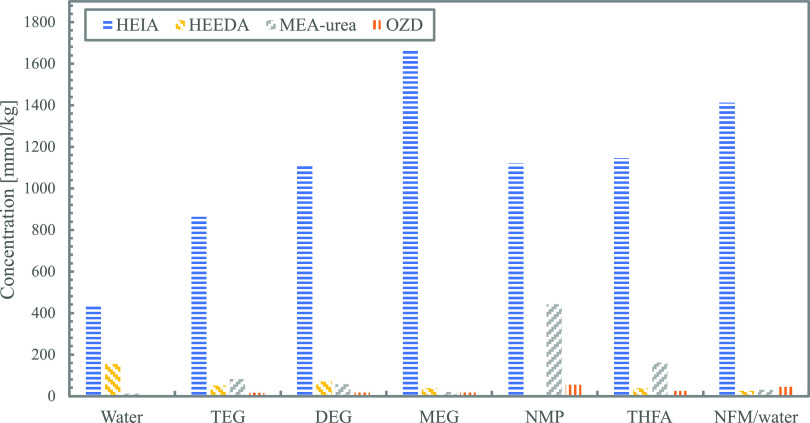
Thermal degradation products formed in solutions of loaded MEA (43 mol %, α = 0.5) in various diluents after 5 weeks.

When comparing this with MEA degraded in the other organic solvents, a similar result can be seen. Figure 15 shows the thermal degradation products formed during thermal degradation of loaded MEA (α = 0.5) in various organic diluents. In all cases when switching water with another diluent, the amount of HEIA produced is nearly doubled. This indicates that it is the removal of water that results in the formation of HEIA being favored. This is especially interesting in the case of the NFM/water mixture, where the amount of HEIA is tripled from only changing 20 mol % water to NFM. Another interesting point is the high formation of MEA urea in the loaded MEA degraded in NMP. It is not clear why these different degradation patterns take place. Overall, from these results, it becomes clear that further studies are needed to understand the degradation mechanisms.

## 4 Conclusions

Water-lean solvents have been proposed as a possible alternative to aqueous amine systems in postcombustion carbon capture. There is however little data available on how amine degradation is affected by different solvents. This study presents new insights on the effect of solvent on thermal degradation of alkanolamines from laboratory-scale degradation experiments.

To investigate the effect of the water on thermal degradation, water was replaced by increasing amount of MEA in the first series of experiments. It was observed that the amine and water concentration did not affect the thermal degradation rate of the amine. An increase in the CO_2_ concentration, however, resulted in increased thermal degradation. Which CO_2_-carrying species is responsible for the increased degradation rates is not clear and it is an interesting topic for further investigations.

In the next experiments, water was replaced with the organic diluent TEG. Solutions of loaded MEA in TEG and water were prepared, and an increased ratio of TEG gave increased thermal degradation of MEA. Experiments with other primary and secondary amines (AP, MMEA, and EAE in TEG) gave the same outcome. It was not concluded whether the stability of tertiary amines (DMMEA, DEEA, and DMPA in TEG) was affected due to their high thermal stability.

Replacing the water in aqueous MEA solutions with organic diluents resulted in varying thermal degradation rates. Overall, all tested organic diluents (DEG, MEG, THFA, NFM/water, and NMP) resulted in higher thermal degradation rates for loaded MEA. None of the proposed parameters such as acid–base behavior, polarity, or relative permittivities, stood out as single contributing factors for the variation in degradation rates. The typical degradation compounds observed for an aqueous MEA solvent were also observed for MEA in various concentrations and with various organic diluents. In conclusion, it seems to be necessary to study each water-lean solvent system separately to rule out amine stability issues. Early-stage testing of new solvent systems is important.

## Supporting Information Available

Additional experimental details and extensive experimental results (PDF)
