# Supplementary material for: Impact of Solvent on the Thermal Stability of Amines
Source: Ind Eng Chem Res. 2022 Oct 19;61(43):16179–92. doi: 10.1021/acs.iecr.2c01934 (PMC9634808; doi:10.1021/acs.iecr.2c01934)
Supplement: Supplementary file 1 — ie2c01934_si_001.pdf [file ie2c01934_si_001.pdf]

Supporting Information

The Impact of the Solvent on the Thermal Stability of Amines

Karen K. Høisæter[a], Solrun J. Vevelstad[b], Lucas Braakhuis[a], Hanna K. Knuutila[a]*

[a] Department of Chemical Engineering, NTNU, NO-7491 Trondheim, Norway

[b] SINTEF Industry, P.O.Box 4760 Torgarden, NO-7465, Norway

* Corresponding author: Hanna.knuutila@ntnu.no

Additional experimental details, and extensive experimental results.

Table S1: Overview of samples with higher RSD.

Week	Solution composition	RSD
6	$5\ \frac{n_{amine}}{kg_{H_2O\ +\ TEG}}$, DMPA in 50mol% TEG in water	2.0%
9	$5\ \frac{n_{amine}}{kg_{H_2O\ +\ TEG}}$, DEEA in water	3.5%
12	$5\ \frac{n_{amine}}{kg_{H_2O\ +\ TEG}}$, DMMEA in water	2.5%

Table S2: Overview of cylinder leakages.

Week	Solution composition
1	30% MEA in THFA
1	30% MEA in NMP
2	30% MEA in MEG
2	30% MEA in DEG
2	30% MEA in TEG
3	30% MEA in MEG
5	90% MEA in water, α = 0.1

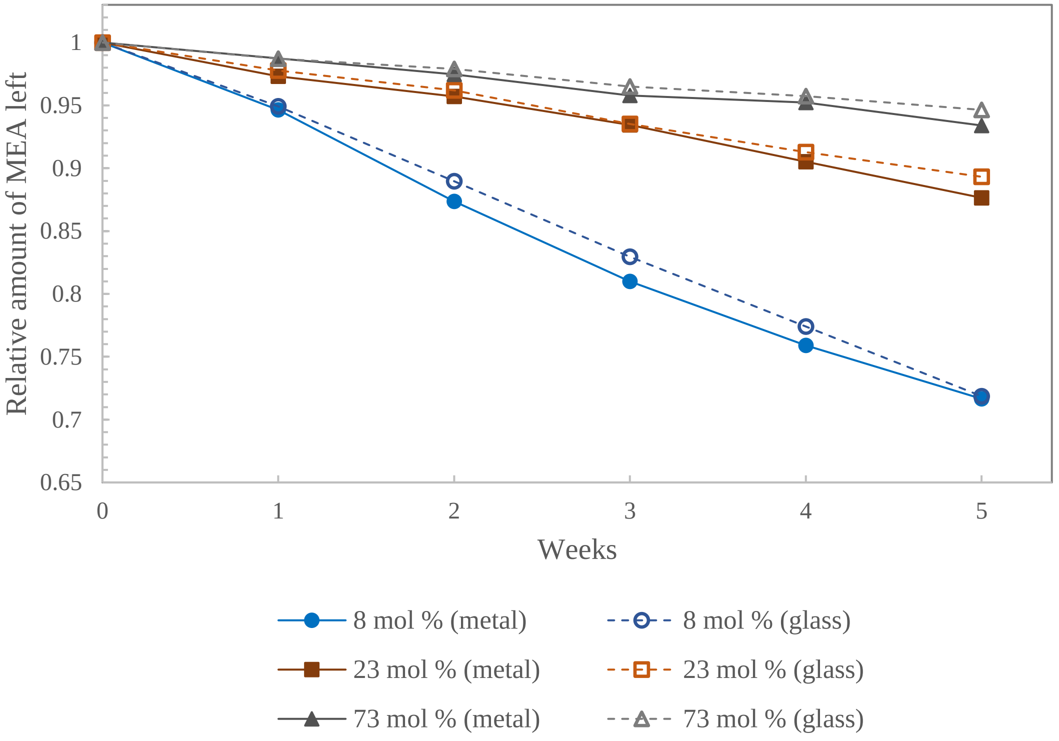

Figure S1: Comparison of thermal degradation results of metal and glass cylinders with loaded MEA (0.19 mol CO2 per 100 g unloaded solution).

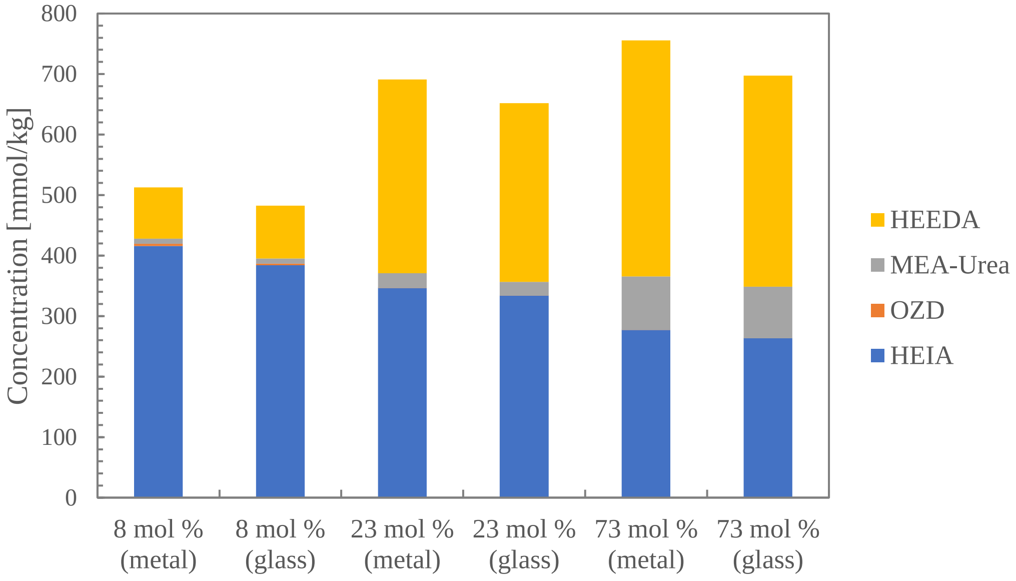

Figure S2: Thermal degradation products of a selection of concentrations of aqueous MEA (0.19 mol CO2 per 100 g unloaded solution) formed after 5 weeks with metal and glass walls.

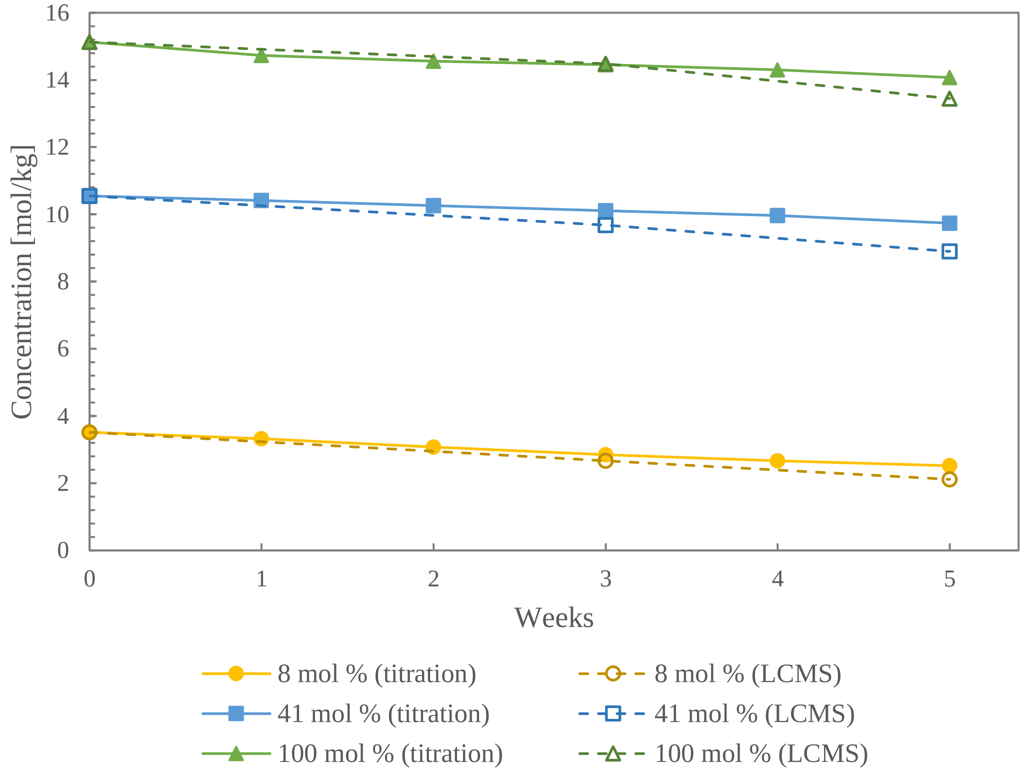

Figure S3: LCMS results vs titration results for comparison.

Table S3: Titration results [mol/kg] for thermal degradation experiments of aqueous MEA conducted in metal cylinders at 135°C.

Week	***0***	***1***	***2***	***3***	***4***	***5***
**11mol% MEA α=0.1**	4.81	4.75	4.74	4.77	4.72	4.75
**11mol% MEA α=0.2**	4.71	4.64	4.63	4.54	4.46	4.47
**11mol% MEA α=0.3**	4.62	4.51	4.42	4.25	4.03	4.09
**11mol% MEA α=0.4**	4.56	4.35	4.12	3.87	3.66	3.42
**8mol% MEA α=0.5**	3.52	3.33	3.07	2.85	2.67	2.52
**23mol% MEA α=0.23**	7.57	7.37	7.24	7.07	6.85	6.63
**41mol% MEA α=0.17**	10.55	10.41	10.26	10.11	9.97	9.74

**73mol% MEA α=0.13**	12.95	12.78	12.62	12.40	12.33	12.09
**100mol% MEA α=0.12**	15.13	14.73	14.55	14.45	14.30	14.07
**23mol% MEA α=0.1**	7.93	7.85	7.78	7.77	7.71	7.73
**41mol% MEA α=0.1**	10.87	10.77	10.72	10.73	10.65	10.65
**73mol% MEA α=0.1**	13.75	13.53	13.49	13.36	13.28	13.34

Table S4: Titration results [mol/kg] for thermal degradation experiments of aqueous MEA conducted in cylinders with glass walls at 135°C.

Week	***0***	***1***	***2***	***3***	***4***	***5***
**8mol% MEA α=0.5**	3.52	3.34	3.13	2.92	2.72	2.53
**23mol% MEA α=0.23**	7.57	7.40	7.28	7.08	6.91	6.76
**73mol% MEA α=0.13**	12.95	12.78	12.67	12.49	12.39	12.25

Table S5: Titration results [mol/kg] for thermal degradation experiments of MEA (5 $\frac{n_{MEA}}{kg_{H_2O+TEG}}$, α=0.5) in TEG and water conducted in metal cylinders at 135°C.

Week	***0***	***1***	***2***	***3.1***	***4***	***5***
**Water**	3.52	3.33	3.07	2.84	2.67	2.49
**5mol% TEG**	3.58	3.36	3.10	2.81	2.64	2.38
**20mol% TEG**	3.60	3.25	2.94	2.59	2.40	2.11
**50mol% TEG**	3.56	3.09	2.73	2.41	2.22	1.92
**80mol% TEG**	3.58	3.00	2.57	2.22	2.02	1.78
**TEG**	3.54	2.88	2.42	2.04	1.88	1.62

Table S6: Titration results [mol/kg] for thermal degradation experiments of primary amine AP and secondary amines MMEA and EAE in water and TEG (5 $\frac{n_{MEA}}{kg_{H_2O+TEG}}$, α=0.5) conducted in metal cylinders at 135°C.

Week	***0***	***1***	***2***	***3***	***4***	***5***
**AP in water**	3.36	3.24	3.20	3.17	3.12	3.12
**MMEA in water**	3.27	2.97	2.72	2.64	2.46	2.45
**EAE in water**	2.90	2.75	2.58	2.51	2.46	2.46
**AP in 50mol% TEG**	3.34	2.76	2.30	2.10	1.89	1.90
**MMEA in 50mol% TEG**	3.32	2.88	2.58	2.47	2.31	2.30
**EAE in 50mol% TEG**	3.08	2.59	2.37	2.25	2.18	2.18

Table S7: Titration results [mol/kg] for thermal degradation experiments of tertiary amines DMMEA, DEEA and DMPA in water and TEG (5 $\frac{n_{MEA}}{kg_{H_2O+TEG}}$, α=0.3) conducted in metal cylinders at 135°C.

Week	***0***	***2***	***4***	***6***	***9***	***12***
**DMMEA in water**	3.02	2.99	3.08	2.96	2.97	2.94
**DEEA in water**	2.60	2.58	2.57	2.54	2.56	2.46
**DMPA in water**	2.93	2.96	2.93	2.96	2.89	2.85
**DMMEA in 50mol% TEG**	3.00	3.01	2.99	2.98	2.94	2.93
**DEEA in 50mol% TEG**	2.95	3.08	2.95	2.94	2.90	2.89
**DMPA in 50mol% TEG**	2.98	2.95	2.96	3.07	2.95	2.97

Table S8: Titration results [mol/kg] for thermal degradation experiments of MEA (43mol%, α=0.5) in organic diluents conducted in metal cylinders at 135°C.

Week	***0***	***1***	***2***	***3***	***4***	***5***
**Water**	3.52	3.33	3.07	2.84	2.67	2.49
**MEG**	5.94	4.10	2.95	2.10	1.65	1.28

**DEG**	4.42	3.66	3.13	2.58	2.23	1.93
**TEG**	3.60	2.88	2.42	2.04	1.88	1.62
**20mol% NFM in water**	7.95	6.60	5.54	4.46	3.97	3.51
**NMP**	4.38	2.73	2.37	1.90	1.62	1.58
**THFA**	4.39	3.47	3.12	2.58	2.36	2.21

Table S9: LCMS results [mmol/kg] for thermal degradation experiments of aqueous MEA in metal cylinders at 135°C.

Mol%	***α***	***Week***	***MEA***	***HEIA***	***OZD***	***MEA-urea***	***HEEDA***
**11**	**0.1**		4823	14.5	0.47	5.36	29.7
**11**	**0.2**	**3**	4368	53.9	0.57	10.33	88.9
**11**	**0.3**		3991	152.6	1.27	14.50	128.8
**11**	**0.1**		4807	30.8	0.58	5.47	56.5
**11**	**0.2**	**4**	4238	88.5	0.70	9.66	98.4
**11**	**0.3**		3525	258.9	0.76	11.91	138.3
**8**	**0.5**		2670	266.2	4.76	12.29	94.2
**41**	**0.17**	**3**	9676	153.4	0.11	56.37	274.2
**100**	**0.12**		14482	164.5	0.06	203.13	253.4
**8**	**0.5**		2116	415.8	3.87	8.47	84.6
**11**	**0.4**		2894	431.4	2.07	11.41	154.4
**23**	**0.23**		5803	346.3	0.23	24.48	319.8
**41**	**0.17**	**5**	8899	279.6	0.09	47.70	373.0
**73**	**0.13**		11332	277.1	0.05	88.25	390.1
**100**	**0.12**		13447	330.7	0.02	178.69	378.3
**23**	**0.1**		7331	55.7	< 0.13	16.00	142.4
**41**	**0.1**	**4**	10568	67.2	< 0.13	37.06	188.1
**73**	**0.1**		12983	124.5	< 0.13	108.46	216.1

Table S10: LCMS results [mmol/kg] for thermal degradation experiments of MEA (5 $\frac{n_{MEA}}{kg_{H_2O+TEG}}$, α=0.5) in TEG and water in metal cylinders at 135°C.

Mol% TEG	***Week***	***MEA***	***HEIA***	***OZD***	***MEA-Urea***	***HEEDA***
**50**	**3**	2203	476.7	14.01	92.99	75.2
**100**		1946	599.2	19.7	131.22	61.4
**5**	**5**	2056	469	5.63	15.93	81.1
**20**		1785	668.1	8.46	37.14	72.4
**50**		1708	756.2	11.1	60.43	64.0
**80**		1592	829.6	14.91	84.89	57.6
**100**		1397	863.6	14.54	81.38	51.1

Table S11: LCMS results [mmol/kg] for thermal degradation experiments of MEA (43mol%, α=0.5) in organic diluents in metal cylinders at 135°C.

Solvent	***Week***	***MEA***	***HEIA***	***OZD***	***MEA-urea***	***HEEDA***
**TEG**	**3**	1946	599.2	19.7	131.22	61.4
**DEG**		2188	818.5	22.29	102.46	94.83
**MEG**		1356	1486.8	22.98	56.44	83.41
**NMP**		1829	729.8	60.58	587.22	1.47
**THFA**		2427	821.5	28.84	217.59	50.06
**20mol%NFM in water**		1861	1102.5	45.40	71.32	53.68
**TEG**	**5**	1397	863.6	14.54	81.38	51.09
**DEG**		1519	1115.0	17.31	56.70	69.37
**MEG**		613	1671.2	16.48	18.89	38.35
**NMP**		1400	1119.6	54.21	441.11	0.52
**THFA**		1973	1144.7	24.28	161.23	38.07
**20mol% NFM in water**		904	1412.0	42.87	30.66	25.63

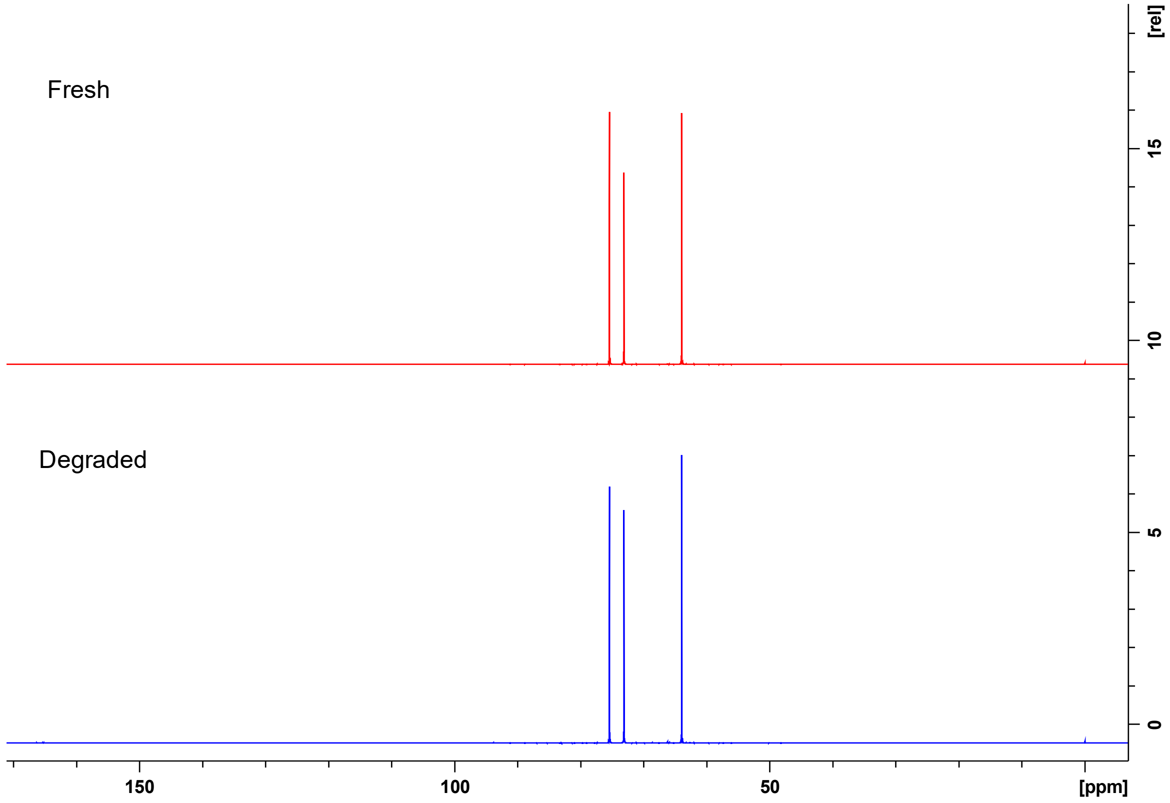

Figure S4: NMR spectra for comparison of pure TEG thermally degraded for 5 weeks and fresh, undegraded TEG.

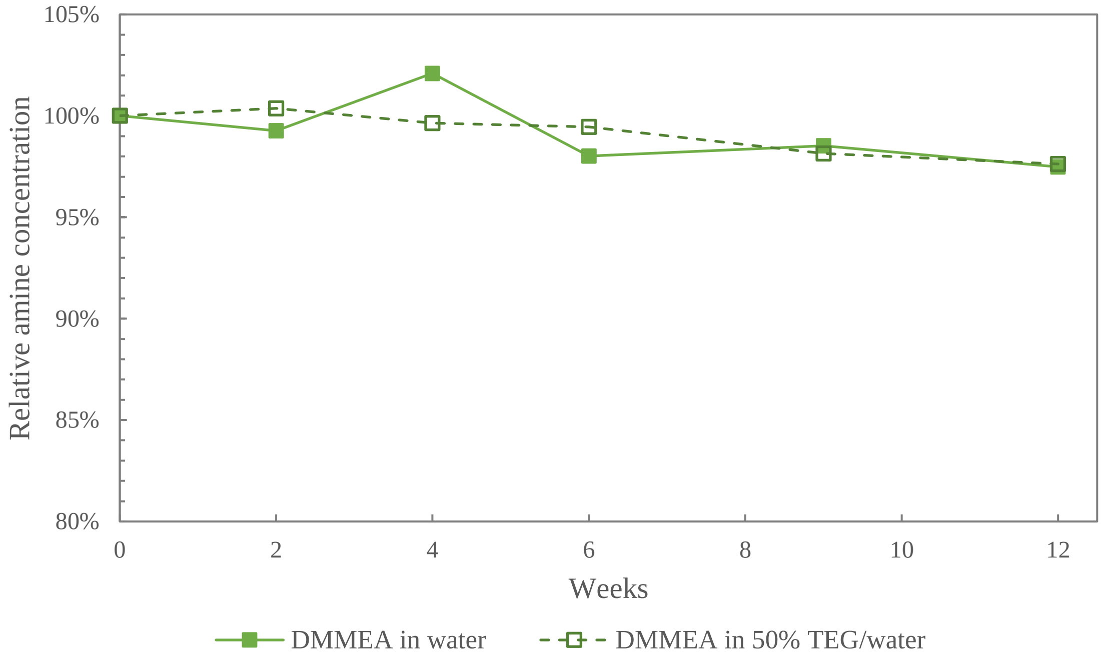

Figure S5: Effect of addition of TEG on thermal stability of tertiary amine DMMEA (5 $\frac{n_{amine}}{kg_{H_2O+TEG}}$, α = 0.3, 135 °C).

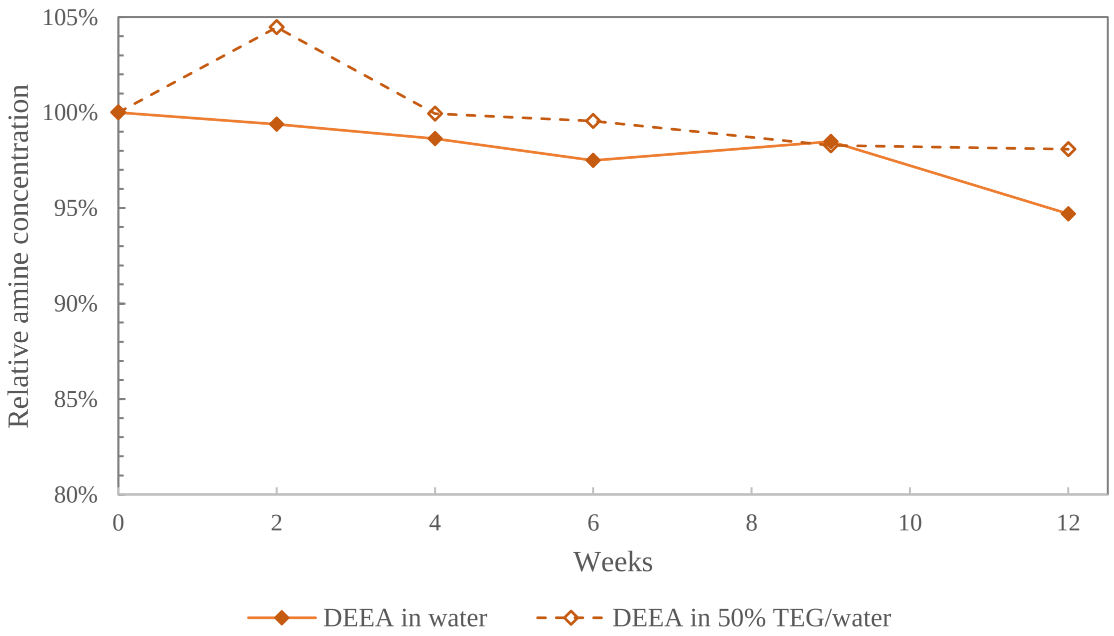

Figure S6: Effect of addition of TEG on thermal stability of tertiary amine DEEA (5 $\frac{n_{amine}}{kg_{H_2O+TEG}}$, α = 0.3, 135 °C).

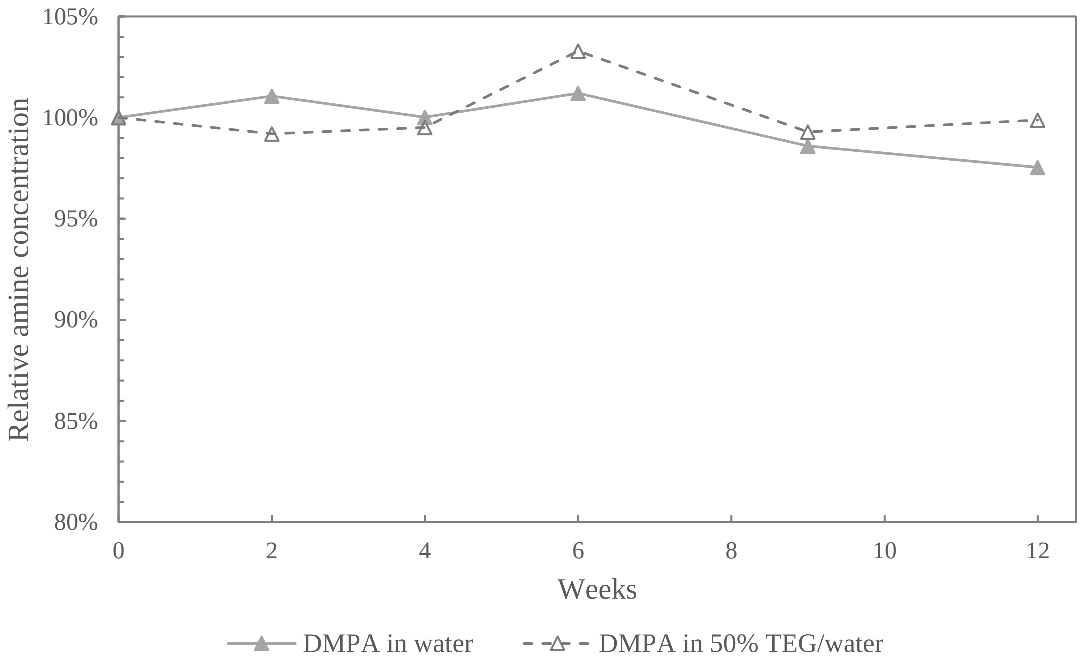

Figure S7: Effect of addition of TEG on thermal stability of tertiary amine DMPA ($5\ \frac{n_{amine}}{kg_{H_2O\ +\ TEG}}$*, α = 0.3, 135 °C).*
